# A nomogram integrating YTHDC2 expression and clinicopathological parameters for predicting endometrial cancer recurrence: a multicenter retrospective study

**DOI:** 10.3389/fonc.2026.1930179

**Published:** 2026-10-19

**Authors:** Yu Gong, Lamei Hou, Jie Xiong, Chenfan Tian, Yuan Tu, Peng Jiang, Rui Yuan

**Affiliations:** 1 The First Affiliated Hospital of Chongqing Medical University, Chongqing, China; 2 Department of Gynecology, Suining Central Hospital, Suining, China; 3 Department of Gynecology, Fengdu General Hospital, Chongqing, China; 4 Department of Gynecology, Liangjiang Hospital of Chongqing Medical University, Chongqing, China

**Keywords:** endometrial cancer, nomogram, recurrence, training cohort, validation cohort, YTHDC2

## Abstract

**Background:**

Endometrial cancer (EC) is one of the most common gynecological malignancies worldwide, with persistently rising incidence and mortality. Reliable molecular biomarkers for predicting EC recurrence remain lacking. This study aimed to develop a nomogram integrating YTHDC2 expression with clinicopathological parameters to predict postoperative recurrence-free survival (RFS) in EC patients.

**Methods:**

A total of 520 stage I–III EC patients undergoing initial standard surgical treatment were enrolled as the training cohort, and 243 patients from three independent hospitals constituted the validation cohort. Univariate and multivariate Cox regression analyses were performed to identify independent prognostic factors for RFS in the training cohort, and a nomogram was constructed accordingly. Internal validation was conducted using the concordance index (C-index) and calibration curves in the training cohort, and external validation was performed in the validation cohort. Risk stratification was based on the Nomogram-predicted 3-year RFS probability, and Kaplan–Meier curves were used to evaluate the clinical utility of the model. The nomogram’s discriminative performance was further compared with three published EC prediction models.

**Results:**

Univariate and multivariate Cox regression analyses identified age (HR = 2.02, *P* = 0.006), International Federation of Gynecology and Obstetrics (FIGO) stage (HR = 4.20, *P* < 0.001), lymphovascular space invasion (LVSI; HR = 1.81, *P* = 0.022), CA125 level (HR = 1.66, *P* = 0.048), depth of myometrial invasion (HR = 1.68, *P* = 0.039), histological type (HR = 3.42, *P* < 0.001), p53 protein status (HR = 2.61, *P* < 0.001), and YTHDC2 high expression level (HR = 0.26, *P* < 0.001) as independent prognostic factors for RFS. A nomogram incorporating these eight factors was constructed to predict RFS in EC. Internal and external calibration curves demonstrated good agreement between predicted and observed outcomes. The model achieved a C-index of 0.892 in the training cohort and 0.859 in the validation cohort, significantly outperforming single-indicator models. It also surpassed three published EC prediction models in both cohorts. The nomogram consistently outperformed single-indicator models in predicting 1-, 3-, and 5-year RFS.

**Conclusion:**

The nomogram integrating YTHDC2 expression with clinicopathological parameters demonstrated favorable accuracy in predicting RFS for patients with EC and may effectively guide long-term prognostic management.

## Introduction

Endometrial cancer (EC) is the most common gynecological malignancy in developed countries, accounting for roughly 5.9% of all cancers in women and exceeding 400,000 new cases annually worldwide (1, 2). Standard treatment for operable EC consists of total hysterectomy with bilateral salpingo-oophorectomy and lymph node assessment. Despite generally favorable responses to primary therapy in early-stage disease, tumor heterogeneity drives an overall recurrence rate of 15–20% even after complete surgical staging and adjuvant radiotherapy or chemotherapy (3–5). Recurrent EC frequently exhibits treatment resistance and is closely tied to elevated all-cause mortality (6). A fundamental obstacle is the absence of validated molecular biomarkers for recurrence prediction, which limits both risk-adapted treatment and prognostic precision (7–9). Molecular feature-based risk stratification models have shown early promise (10, 11), yet clinical translation of key targets remains elusive. Reliable biomarkers are therefore needed to build individualized models that can meaningfully inform EC treatment outcomes and prognosis.

Recommendations for adjuvant therapy after EC surgery rest largely on clinicopathological variables — age, disease stage, histological subtype and grade, depth of myometrial invasion, and the presence of lymphovascular space invasion (LVSI) (12). The 2023 International Federation of Gynecology and Obstetrics (FIGO) staging system (13)adopts a four-tier classification grounded in the TNM framework and incorporates histological type and molecular subtype. These subtypes were refined in the Proactive Molecular Risk Classifier for Endometrial Cancer (ProMisE), which uses immunohistochemical assessment of p53 and mismatch repair proteins together with targeted POLE sequencing to classify EC into four categories: p53 wild-type, MMR-deficient, p53 abnormal, and POLE-mutant (14). Molecular classification carries prognostic weight, and NCCN guidelines now recommend it for all patients with newly diagnosed EC (15). That said, the existing system is limited by cost and suboptimal diagnostic performance, underscoring the demand for auxiliary markers that sharpen risk stratification and support clinical decisions.

N6-methyladenosine (m^6^A) modification and its reader proteins regulate RNA splicing, stability, and translation, and their dysregulation contributes to tumor progression. Among the m^6^A readers, YTHDC2 has drawn particular interest for its tumor-suppressive roles across multiple cancer types; pan-cancer analyses link high YTHDC2 expression to better chemotherapy response, prolonged survival, and an immunologically active tumor microenvironment (16, 17).Within endometrial cancer, we performed a systematic screen of m^6^A regulators in public transcriptomic data, in which YTHDC2 ranked among the most consistently down-regulated(**Supplementary Figure 1**), and prognostically relevant candidates (**Supplementary Figure 2**), providing an evidence-based rationale for its prioritisation over other m^6^A readers.

Earlier work in EC supports this choice: Wu et al (18) identified YTHDC2 as one of six regulators contributing to a risk signature in endometrial adenocarcinoma, and Zhang et al (19) reported reduced YTHDC2 expression in EC tissues, its correlation with myometrial invasion, lymph node metastasis, and FIGO stage, and its suppression of EC progression through the Hedgehog pathway. These studies nonetheless relied on transcriptomic or single-centre data and did not translate YTHDC2 into a clinically applicable, externally validated prediction tool—the gap the present study addresses.

Here we quantified YTHDC2 protein by immunohistochemistry—rather than mRNA—in a multicenter cohort, integrated it with established clinicopathological parameters, and built a nomogram that estimates recurrence-free survival (RFS). The model was externally validated in three independent centres. This approach moves YTHDC2 from a transcriptomic prognostic association toward a practical, immunohistochemistry-based tool for individualized postoperative management in EC.

## Materials and methods

### Study population

This multicenter retrospective cohort study enrolled patients with stage I–III EC who underwent primary standard surgical treatment between January 2018 and January 2023. The training cohort comprised 520 patients from the First Affiliated Hospital of Chongqing Medical University. An independent external validation cohort of 243 patients was drawn from three centers: Suining Central Hospital (n=101), the Liangjiang Branch of Chongqing Medical University (n=78), and Fengdu County People’s Hospital (n = 64).

Patients were enrolled if they had pathologically confirmed stage I–III EC (FIGO 2009 criteria), underwent standardized surgery defined as total hysterectomy with bilateral adnexectomy and systematic lymph node assessment when indicated by pathological characteristics (20), and had complete baseline clinical records, standard preoperative laboratory tests (including tumor markers such as CA125), and imaging evaluation (chest and abdominal CT, pelvic MRI). Reasons for exclusion were: not receiving standard surgery, neoadjuvant therapy before surgery, incomplete medical documentation, history of another malignancy, and loss to follow-up. The patient selection flow is illustrated in **Figure 1**.

**Figure 1:**
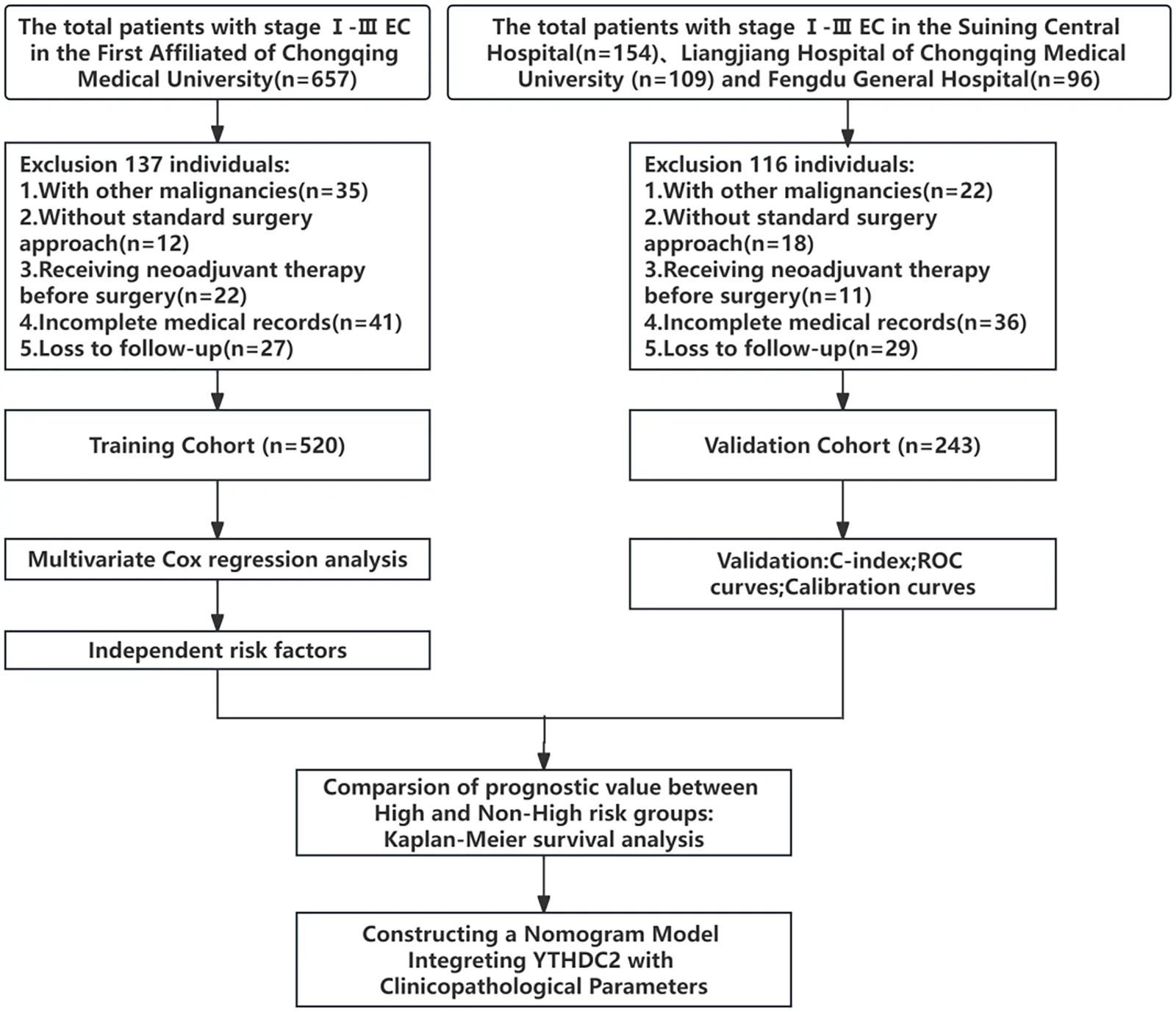
Flowchart of patient selection and cohort allocation.

### Surgical treatment and adjuvant therapy

All patients underwent total hysterectomy, bilateral salpingo-oophorectomy, and lymph node staging (sentinel lymph node mapping with or without pelvic and/or para-aortic lymphadenectomy). Adjuvant treatment was individualized according to postoperative histopathology, molecular features, and recurrence risk factors. Factors linked to higher recurrence risk in endometrioid carcinoma include older age, LVSI, higher tumor grade, and deep myometrial invasion (21). Patients who met high- or intermediate-risk criteria derived benefit from postoperative vaginal brachytherapy (22); patients with early-stage endometrioid carcinoma lacking high- or intermediate-risk features were managed with surveillance (23).

### Postoperative follow-up protocol

Patients were followed according to a standardized schedule: every 3–6 months for the first 2 postoperative years, every 6 months in years 3–5, and annually thereafter. Visits included a structured history focusing on symptoms suggestive of recurrence, a pelvic examination to assess the vaginal cuff and pelvis, and — when clinically indicated — laboratory and imaging studies. Routine serial CA125 monitoring was not recommended for all patients; it was reserved for cases with initially elevated CA125 and extrauterine or advanced-stage disease, aggressive histological types (serous or clear cell carcinoma), or clinical suspicion of recurrence. Imaging (pelvic ultrasound, CT, MRI, FDG-PET/CT, or chest X-ray) was performed selectively when symptoms or physical findings raised concern. Follow-up was censored in February 2026. Because approximately 80% of EC recurrences manifest within 3 years of surgery, all surviving patients completed at least 36 months of follow-up, unless lost to follow-up or deceased (5).

Recurrence was defined as the first post-surgical documentation of disease recurrence or metastasis, confirmed pathologically or by unequivocal imaging evidence. The diagnosis required independent agreement by at least two gynecologic oncology specialists. Persistently elevated serum tumor markers (e.g., CA125) after excluding alternative explanations served as supportive, rather than definitive, evidence for recurrence (24).

RFS was defined as the interval from the date of definitive surgical staging to the first confirmed recurrence, recurrence-related death, or last follow-up. Overall survival (OS) was defined as the interval from the date of surgery to death from any cause or last follow-up (25).

### Data collection

Clinicopathological data — including age, height, weight, serum CA125 level, FIGO stage, histological subtype and grade, cervical stromal involvement, depth of myometrial invasion (<1/2 vs. ≥1/2), and LVSI — were collected retrospectively. Paraffin-embedded postoperative tissue specimens were retrieved from the pathology department for immunohistochemical detection of YTHDC2 and p53.

### Data quality control and variable processing

Baseline characteristics, clinicopathological data, and follow-up information were extracted from electronic medical records and independently cross-verified by two investigators. Categorical variables (FIGO stage, histological type) were recoded according to the FIGO 2009 staging system and the WHO 2020 histological classification. Patients with incomplete data on key variables were excluded. The number of patients excluded at each step, stratified by cohort and reason, is summarised in the patient selection flowchart (**Figure 1**). In the training cohort (n = 657), 137 patients (20.9%) were excluded, leaving 520 for analysis; in the external validation cohort (n = 359 from three centres), 116 patients (32.3%) were excluded, leaving 243. The proportion excluded for incomplete medical records on key variables was 6.2% (training) and 10.0% (validation). Given this low proportion of missing data, complete-case analysis was used rather than multiple imputation.

YTHDC2 and p53 immunohistochemical staining was performed centrally at the Department of Pathology, First Affiliated Hospital of Chongqing Medical University, and scored independently by two pathologists under blinded conditions. Cases were excluded if archived tumor tissue was insufficient, IHC staining failed. YTHDC2 expression was evaluated using the semiquantitative scoring system described under Immunohistochemistry. The median score in the training cohort served as the cutoff, with values ≥ 2.5 classified as high expression.

To improve the nomogram’s clinical utility, selected continuous variables were dichotomized using validated, clinically accepted thresholds: age was split at 60 years (≥60 vs. <60), and serum CA125 was split at 35 U/mL. Baseline comparability between training and validation cohorts was assessed with the chi-square test for categorical variables and the t-test for continuous variables. Follow-up was closed on February 28, 2026.

### Tissue processing and pathological evaluation

Postoperative tissue specimens were fixed in formalin, and paraffin embedding, sectioning, and immunohistochemical staining were carried out according to uniform protocols at the pathology departments of the First Affiliated Hospital of Chongqing Medical University, Suining Central Hospital, the Liangjiang Branch of Chongqing Medical University, and Fengdu County People’s Hospital, with the aim of delineating cancerous regions.

Pathological assessment encompassed tumor location, maximum diameter, histological type and grade, depth of myometrial invasion, lymphovascular space invasion (LVSI), and cervical stromal involvement. Histological types were classified as G1/G2 endometrioid adenocarcinoma, G3 endometrioid adenocarcinoma, and non-endometrioid adenocarcinoma (including serous carcinoma, clear cell carcinoma, and other subtypes) (25, 26).

### Immunohistochemistry

Formalin-fixed, paraffin-embedded (FFPE) tumor blocks from the three external validation centers were shipped to the central pathology laboratory at the First Affiliated Hospital of Chongqing Medical University, where all immunohistochemical staining was performed under a unified protocol to eliminate inter-laboratory variation. Serial sections (4 μm) were deparaffinized in xylene, rehydrated through graded ethanol, and subjected to heat-induced antigen retrieval in citrate buffer (pH 6.0) under high pressure. Staining was carried out on an automated IHC platform (Leica Bond-Max, Milton Keynes, UK) using optimized and validated protocols (27).

The primary antibodies were rabbit anti-human YTHDC2 polyclonal antibody (1:1000 dilution; catalog no. 27779-1-AP, Proteintech) and mouse anti-human p53 polyclonal antibody (catalog no. 10442-1-AP, Proteintech), both incubated overnight at 4 °C. PBS substituted for the primary antibody served as a negative control, and known positive tissue sections served as positive controls; both were included in every staining run.

YTHDC2 cytoplasmic staining was semiquantitatively scored by two pathologists blinded to clinical outcomes. Staining intensity was graded on a 4-point scale (1, negative; 2, weak; 3, moderate; 4, strong), and the percentage of positively stained tumor cells (0–100%) was recorded. The final score for each specimen was calculated as staining intensity × the proportion of positive tumor cells (percentage expressed as a decimal from 0 to 1), yielding a continuous score from 0 to 4. Using the median score of the training cohort as the cutoff threshold, specimens with scores ≥ 2.5 were classified into the high-expression group for subsequent nomogram incorporation. Representative IHC images (**Figure 2**) show markedly reduced YTHDC2 staining in recurrent tumors relative to non-recurrent tumors.

**Figure 2:**
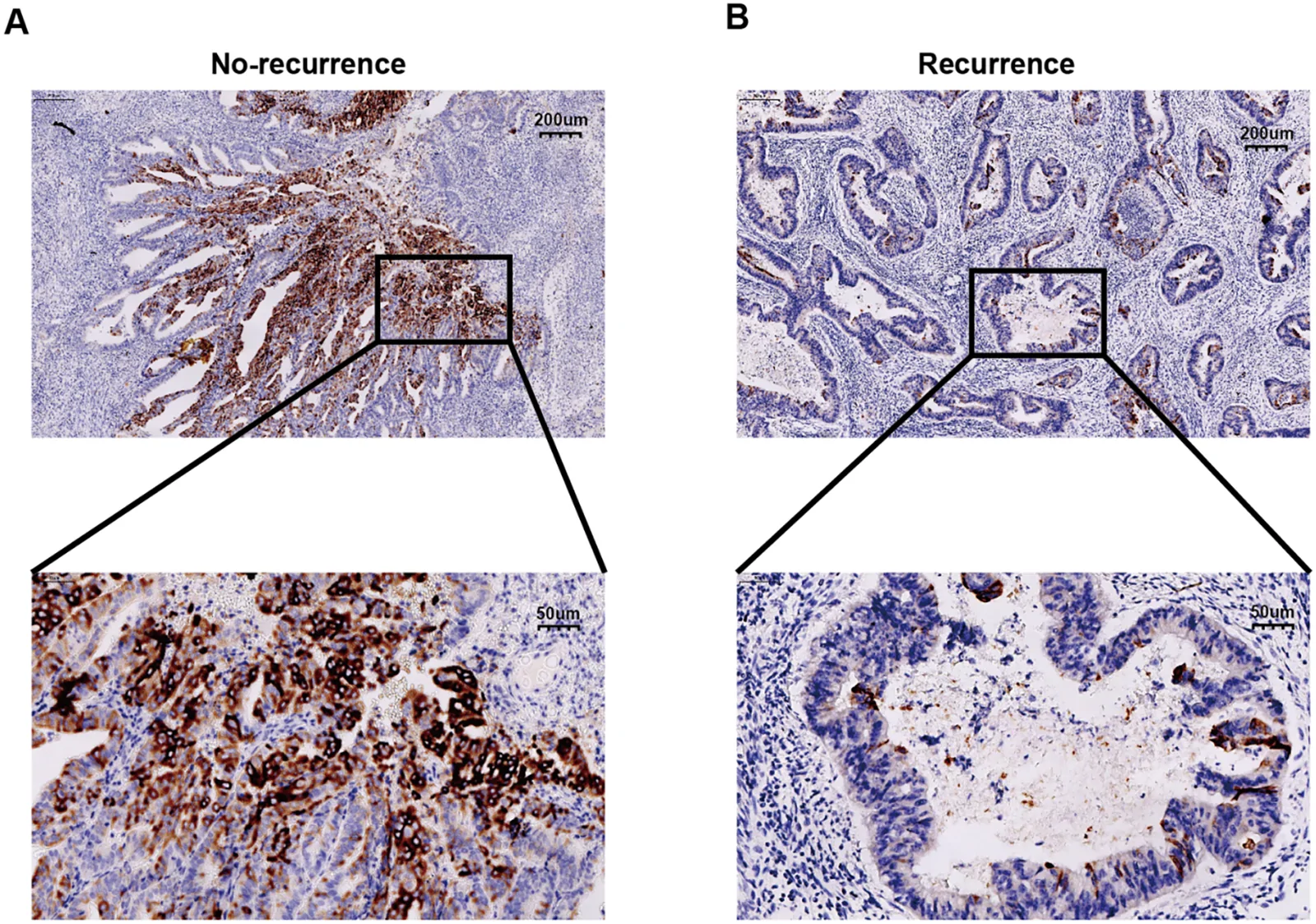
Representative photomicrographs of YTHDC2 immunohistochemical staining. **(A)** High YTHDC2 expression in a case without tumor recurrence. **(B)** Low expression in a case with recurrence.

p53 immunohistochemical expression was evaluated using a three-tier classification(**Supplementary Figure 7**) (28): diffuse strong nuclear staining in ≥80% of tumor cells (overexpression) and complete absence of nuclear staining were both defined as aberrant (mutant-type) p53 expression, whereas staining patterns between these two extremes — an admixture of negative, weakly, and strongly positive cells — were defined as normal (wild-type) p53 expression. Complete absence was recorded only when internal positive controls (fibroblasts, endothelial cells, or lymphocytes) showed intact nuclear staining; cases without a positive internal control were regarded as uninterpretable.

### Statistical analysis

This retrospective cohort study enrolled 520 patients with endometrial cancer from the First Affiliated Hospital of Chongqing Medical University (training cohort) and 243 patients from the Liangjiang Branch of Chongqing Medical University, Suining Central Hospital, and Fengdu County People’s Hospital (external validation cohort), treated between January 2018 and January 2023.Categorical variables are presented as frequency (percentage) and were compared using the chi-square test or Fisher’s exact test, as appropriate. Continuous variables following a normal distribution are expressed as mean ± standard deviation and were analyzed with the independent-samples t-test; those deviating from normality are reported as median (interquartile range) and were compared with non-parametric tests.

In the training cohort, univariate Cox regression was used to screen clinicopathological variables and YTHDC2 expression for association with recurrence-free survival (RFS). Factors with *P* < 0.05 in univariate analysis were entered into a multivariate Cox regression model to identify independent prognostic factors. The proportional hazards assumption of the multivariable model was verified using Schoenfeld residuals (cox.zph function in the survival package, R). These factors were then incorporated into a nomogram to estimate 1-, 3-, and 5-year RFS probabilities.

To address potential overfitting given eight candidate predictors, we additionally performed LASSO-penalised Cox regression and compared the full model against reduced models using the Akaike information criterion (AIC); optimism-corrected concordance indices were derived from 200 bootstrap resamples. As a sensitivity analysis for the YTHDC2 cutoff, alternative percentile thresholds (tertiles) were evaluated. Competing risks were assessed with a Fine–Gray model treating non-cancer death as a competing event. Pairwise comparisons of concordance indices between the nomogram and the three published models were performed with the DeLong test.

Based on the nomogram-predicted 3-year RFS probability, an optimal cutoff was determined by receiver operating characteristic (ROC) curve analysis using the maximum Youden index (sensitivity + specificity − 1). Patients were stratified into high-risk and low-risk groups according to this cutoff. Survival curves were generated with the Kaplan–Meier method and compared using the log-rank test.

Calibration of the nomogram was evaluated in both the training and validation cohorts using calibration curves. Model discrimination was quantified by the time-dependent area under the receiver operating characteristic curve (AUC), whereas overall discriminative performance for censored survival data was assessed by Harrell’s concordance index (C-index). All statistical analyses were performed with SPSS 27.0 and R 4.3.1. A two-sided P < 0.05 was considered statistically significant.

## Results

### Patient characteristics

A total of 763 patients with endometrial cancer (EC) meeting the inclusion criteria were enrolled, including 520 from the First Affiliated Hospital of Chongqing Medical University (training cohort) and 243 from Suining Central Hospital, Liangjiang Hospital of Chongqing Medical University, and Fengdu County People’s Hospital (validation cohort), as shown in **Figure 1**. The median age of the entire cohort was 53 years. FIGO stage distribution was as follows: stage I in 498 patients (65.3%), stage II in 87 (11.4%), and stage III in 178 (23.3%). Deep myometrial invasion was present in 204 patients (26.7%), and high YTHDC2 expression was observed in 494 (64.7%). LVSI was identified in 193 patients (25.3%), cervical stromal invasion in 107 (14.0%), elevated CA125 (>35 U/mL) in 200 (26.2%), and aberrant p53 expression in 210 (27.5%). Regarding adjuvant treatment, 281 patients (36.8%) were managed with postoperative surveillance alone, 240 (31.5%) received radiotherapy only, 21 (2.8%) received chemotherapy only, and 221 (29.0%) received combined chemoradiotherapy. Baseline clinicopathological characteristics — including age, body mass index (BMI), FIGO stage, histological type, tumor grade, cervical stromal invasion, depth of myometrial invasion, LVSI, adjuvant treatment, CA125 level, and YTHDC2 expression — showed no statistically significant differences between the training and validation cohorts (all *P* > 0.05), confirming comparability between the two groups (**Table 1**).

**Table 1:** Demographic and clinical characteristics of patients with EC.

Variables	Whole population [Case(%)]	Training cohort [Case(%)]	Validation cohort [Case(%)]	*P* Value
**Total**	763	520	243	
**Age(years)**	53 (48,60)	53 (48,60)	55(48,59.5)	0.107
**BMI(Kg/m^2^**)	24.24(21.91,27.00)	24.24(21.92,26.71)	24.50(21.90,27.38)	0.485
FIGO stage				
I	498(65.3)	350(67.3)	148(60.9)	0.218
II	87(11.4)	55(10.6)	32(13.2)	
III	178(23.3)	115(22.1)	63(25.9)	
LVSI				
Negative	570(74.7)	397(76.3)	173(71.2)	0.127
Positive	193(25.3)	123(23.7)	70(28.8)	
Serum CA125				
≤35U/mL	563(73.8)	388(74.6)	175 (72)	0.447
>35U/mL	200(26.2)	132(25.4)	68 (28)	
Cervical stromal invasion				
No	656 (86)	449(86.3)	207(85.2)	0.667
Yes	107(14)	71(13.7)	36(14.8)	
Histological type				
G1-G2 Endometrioid	561(73.5)	384(73.9)	177(72.8)	0.648
G3 Endometrioid	77(10.1)	49(9.4)	28(11.5)	
Non-endometrioid	125(16.4)	87(16.7)	38(15.7)	
P53 expression				
Normal	553(72.5)	366(70.4)	187(77)	0.059
Abnormal	210(27.5)	154(29.6)	56(23)	
Myometrial invasion				
<1/2	559(73.3)	388(74.6)	171(70.4)	0.217
≥1/2	204(26.7)	132(25.4)	72(29.6)	
YTHDC2				
Low-expression	269(35.3)	178(34.2)	91(37.4)	0.386
High-expression	494(64.7)	342(65.8)	152(62.6)	
Adjuvant treatment				
Follow-up	281(36.8)	190(36.5)	91(37.4)	0.979
Radiotherapy only	240(31.5)	165(31.7)	75(30.9)	
Chemotherapy only	21(2.7)	15(2.9)	6(2.5)	
Chemoradiotherapy	221(29)	150(28.8)	71(29.2)	
Recurrence				
No	649(85.1)	443(85.2)	206(84.8)	0.880
Yes	114(14.9)	77(14.8)	37(15.2)	

IGO, International Federation of Gynecology and Obstetrics; LVSI, lymphovascular space invasion; CI,confidence interval; HR,hazard ratio; RFS, recurrence-free survival.

Bold values indicate P < 0.05.

### Independent prognostic factors for RFS

Multivariate Cox regression analysis in the training cohort identified the following independent risk factors for recurrence-free survival (RFS) in endometrial cancer (EC): age ≥60 years (HR = 2.02, *P* = 0.006), FIGO stage (HR = 4.20, *P* < 0.001), depth of myometrial invasion (HR = 1.68, P = 0.039), lymphovascular space invasion (HR = 1.81, *P* = 0.022), histological type (HR = 3.42, *P* < 0.001), CA125 ≥35 U/mL (HR = 1.66, *P* = 0.048), and aberrant p53 expression (HR = 2.61, *P* < 0.001). High YTHDC2 expression emerged as an independent protective factor (HR = 0.26, *P* < 0.001). These results are summarized in **Table 2**.The proportional hazards assumption was satisfied for all covariates in the final multivariable Cox model (Schoenfeld residual test: all *P* > 0.05; global *P* = 0.99)(**Supplementary Table 1**).

**Table 2:** Univariate and multivariate cox analyses on variables for the prediction of RFS of patients with EC in training cohort.

Variables	Univariable analyses			Multivariable analyses		
	Hazard ratio	95% CI	*P* value	Hazard ratio	95% CI	*P* value
Age(years) (≥60yearsVS<60years)	3.49	2.228-5.450	<0.001	2.02	1.227-3.333	0.006
BMI(Kg/m^2^) (≥24VS<24)	1.32	0.839-2.087	0.228	–	–	–
FIGO stage						
I			<0.001	–	–	<0.001
II	5.82	2.608-12.995	<0.001	3.00	0.87-10.33	0.082
III	16.53	9.001-30.348	<0.001	4.20	2.064-8.54	<0.001
LVSI						
Negative VS Positive	3.60	2.305-5.636	<0.001	1.81	1.090-2.991	0.022
Serum CA125						
>35 VS ≤35	3.56	2.278-5.573	<0.001	1.66	1.004-2.735	0.048
Myometrial invasion						
≥1/2 VS <1/2	4.55	2.898-7.152	<0.001	1.68	1.028-2.746	0.039
Cervical stromal invasion						
Yes VS No	2.09	1.231-3.538	0.006	0.90	0.381-2.131	0.812
Histological type						
G1-G2 Endometrioid			<0.001	–	–	<0.001
G3 Endometrioid	3.23	1.501-6.951	<0.001	2.29	1.023-5.125	0.044
Non-endometrioid	10.81	6.566-17.810	<0.001	3.42	1.953-5.995	<0.001
P53 expression						
Abnormal Vs Normal	4.79	3.009-7.623	<0.001	2.61	1.581-4.295	<0.001
YTHDC2						
High-expression VS Low-expression	0.25	0.157-0.400	<0.001	0.26	0.159-0.427	<0.001

BMI, body mass index; FIGO, International Federation of Gynecology and Obstetrics; LVSI, lymphovascular space invasion.

Among 520 patients in the training cohort, death without prior recurrence occurred in only 1 patient (0.19%). In the Fine-Gray competing-risk regression, the subdistribution hazard ratios for all eight predictors were nearly identical to the main Cox estimates (all *P* < 0.05), indicating that the RFS-based conclusions are robust to the competing-risk setting(**Supplementary Table 2**).

### Sensitivity analysis for the YTHDC2 cutoff

We repeated the survival analysis using tertiles of the continuous YTHDC2 score (cutpoints 2.47 and 3.16, derived from the training cohort). In the training cohort, 3-year RFS was 72.4%, 92.4%, and 93.6% for the low, middle, and high tertiles (test for linear trend: χ² = 26.72, df = 1, *P* < 0.001); in the validation cohort, 72.7%, 92.7%, and 94.3% (χ² = 12.53, df = 1, *P* < 0.001). The lower tertile cutpoint (2.47) was nearly identical to the median threshold (2.5), supporting the validity of the dichotomisation. These results show a robust dose–response relationship rather than an artefact of an arbitrary cutoff(**Supplementary Figure 3**, **Supplementary Table 3**).

### Association between YTHDC2 and p53 expression

In the training cohort (n = 520), YTHDC2 expression (high vs. low) was not significantly associated with p53 status (normal vs. abnormal) by the chi-square test (*P* = 0.645), and Spearman’s rank correlation was negligible (ρ = 0.020, *P* = 0.645)(**Supplementary Table 4**). Variance inflation factors for all predictors in the multivariable model ranged from 1.07 to 2.05, confirming the absence of multicollinearity(**Supplementary Table 5**). Representative serial sections demonstrated cytoplasmic YTHDC2 staining and nuclear p53 staining within the same tumour areas(**Supplementary Figure 4**).

### Nomogram construction and validation

A nomogram for predicting 1-, 3-, and 5-year recurrence-free survival (RFS) in endometrial cancer (EC) was constructed by integrating YTHDC2 expression with the independent prognostic factors identified in the multivariate Cox regression analysis (age, FIGO stage, depth of myometrial invasion, lymphovascular space invasion, histological type, CA125 level, and p53 status). The total nomogram score — the sum of points assigned to each predictor — quantitatively estimates individual RFS probabilities (**Figure 3**).

**Figure 3:**
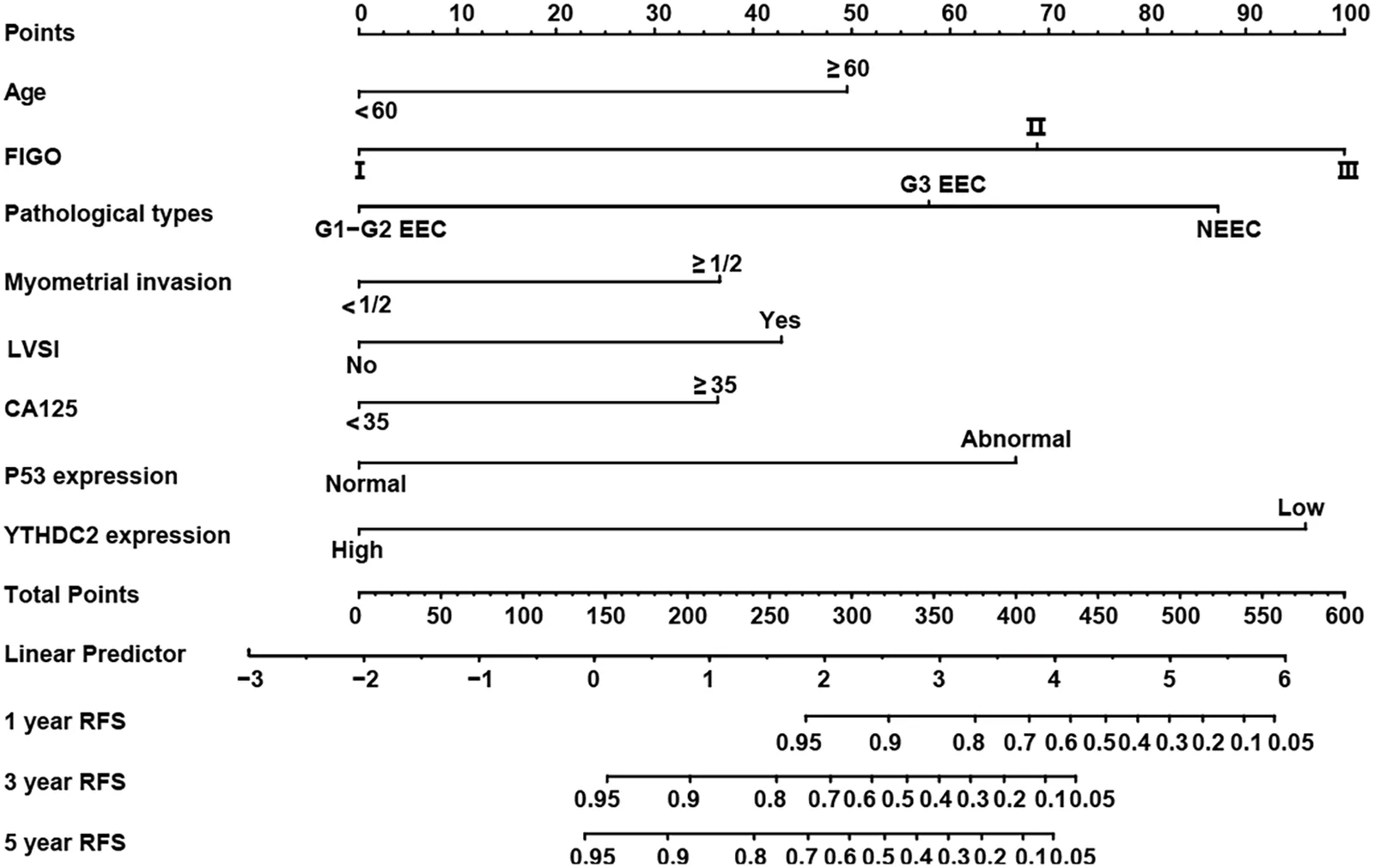
The nomogram for predicting 1-, 3-, and 5-year RFS in EC patients.

The discriminative ability of the nomogram was assessed by ROC curve analysis in both cohorts (**Figure 4**). In the training cohort, the nomogram achieved an area under the ROC curve (AUC) of 0.887 (95%CI:0.844–0.929), exceeding the clinicopathological-only model (AUC = 0.823; 95% CI: 0.770–0.876; *P* = 0.001) and YTHDC2 alone (AUC = 0.680; 95% CI: 0.614–0.747; *P* < 0.001). In the validation cohort, the nomogram similarly outperformed both comparators, with an AUC of 0.863 (95%CI:0.795–0.930) versus 0.811 (95%CI:0.735–0.886) for the clinicopathological model and 0.678 (95% CI: 0.583–0.772) for YTHDC2 alone. The time-dependent AUCs of the nomogram for predicting RFS at 1, 3, and 5 years are presented in(**Supplementary Figure 5**).

**Figure 4:**
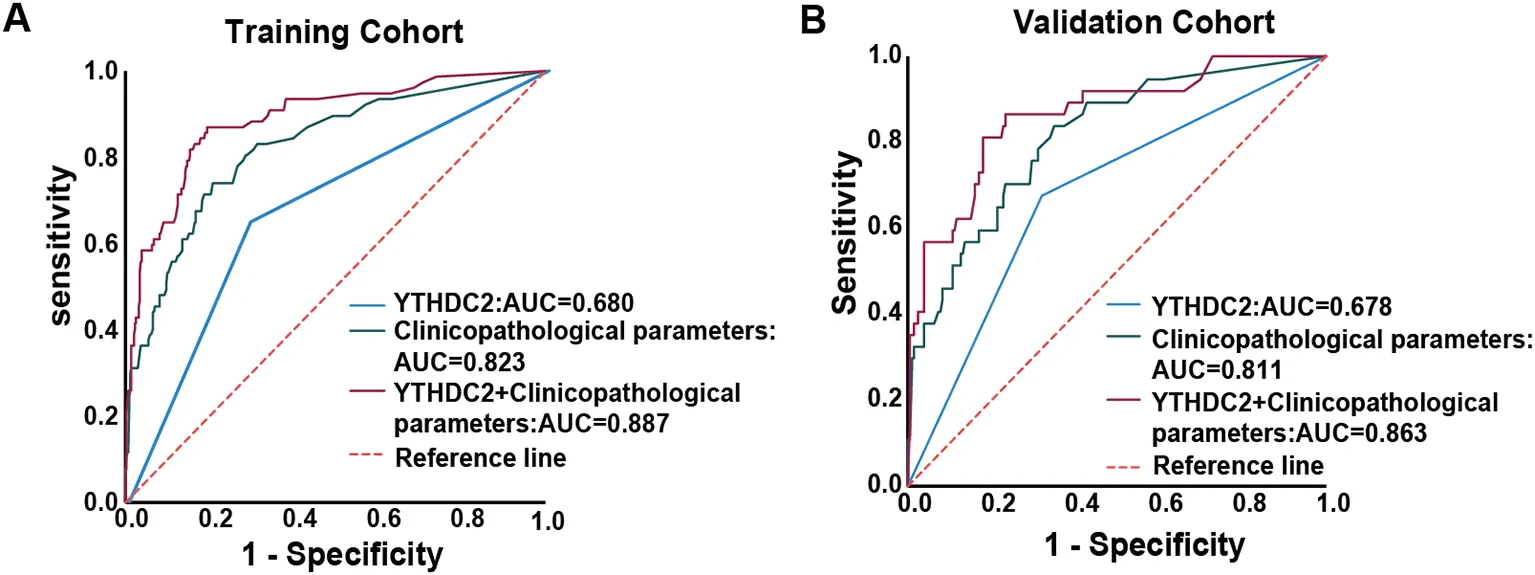
The ROC curves of YTHDC2 alone, individual clinicopathological parameters alone,and their combination in the training cohorts **(A)** and validation cohorts **(B)**.

Calibration curves demonstrated close agreement between nomogram-predicted and observed RFS probabilities in both the training and validation cohorts, indicating satisfactory calibration of the model (**Figure 5**).

**Figure 5:**
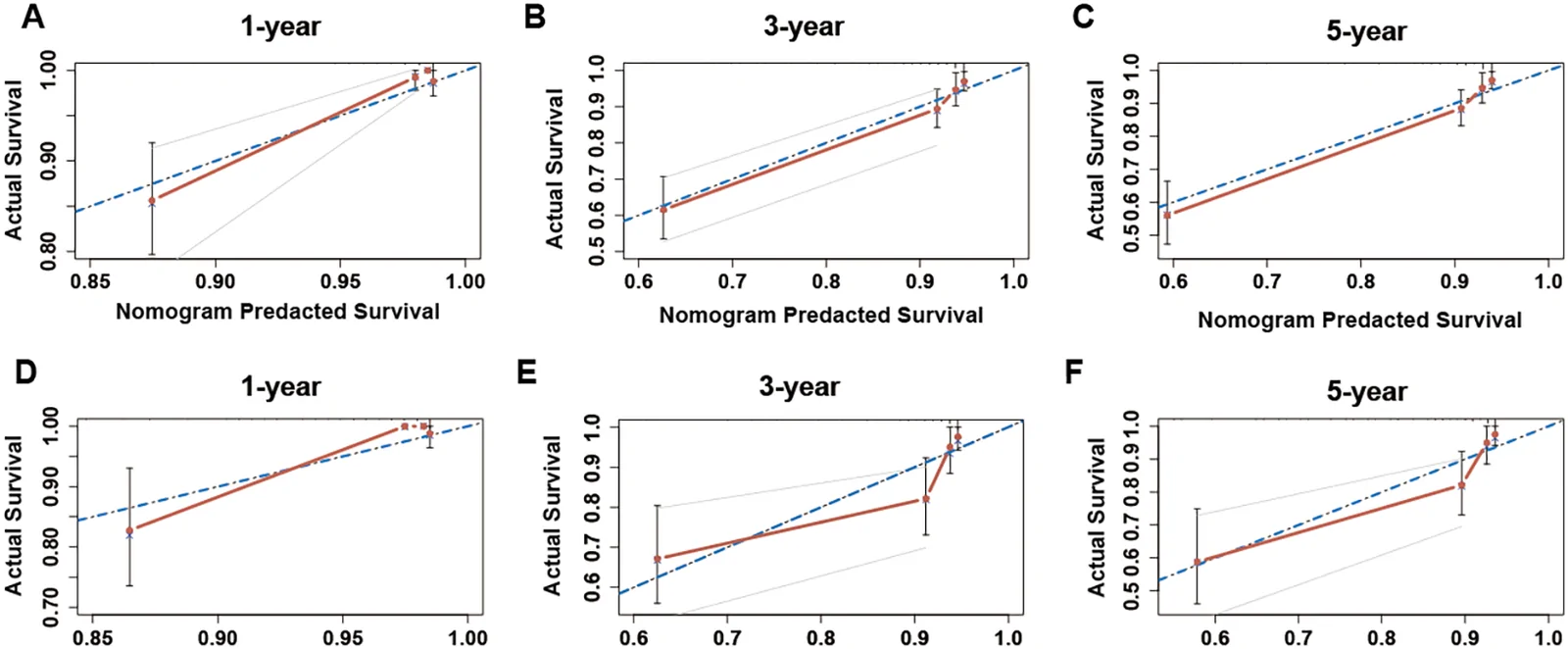
The calibration curves of the nomogram. Calibration curves of 1-, 3-, 5-year RFS in the training set **(A–C)**; and the validation set **(D–F)**.

To assess the stability of the eight-predictor model, we performed LASSO-penalised Cox regression, AIC-based backward stepwise selection, and bootstrap internal validation. LASSO retained all eight predictors at the optimal penalty, and backward stepwise selection based on AIC likewise retained all eight variables(**Supplementary Figure 6**); the full model (AIC = 750.3) outperformed all reduced models, and removal of YTHDC2 increased the AIC by 29.4. Internal validation by 200 bootstrap resamples yielded an optimism-corrected Harrell’s concordance index (C-index) of 0.880, close to the apparent value of 0.892, indicating minimal overfitting.

### Risk stratification and survival analysis

Since most EC recurrences occur within 3 years of surgery, we computed individual 3-year RFS probabilities from the nomogram for each patient in the training cohort. Receiver operating characteristic (ROC) curve analysis was then performed to determine the optimal cutoff for the nomogram-predicted 3-year RFS probability. The cutoff was chosen by maximizing the Youden index (sensitivity + specificity − 1), which yielded a threshold of 0.877 (maximum Youden index, 0.676) (**Figure 6**).Patients with a predicted 3-year RFS probability ≥0.877 were assigned to the low-risk group, and those with values below this cutoff to the high-risk group; this threshold was subsequently applied to the validation cohort.

**Figure 6:**
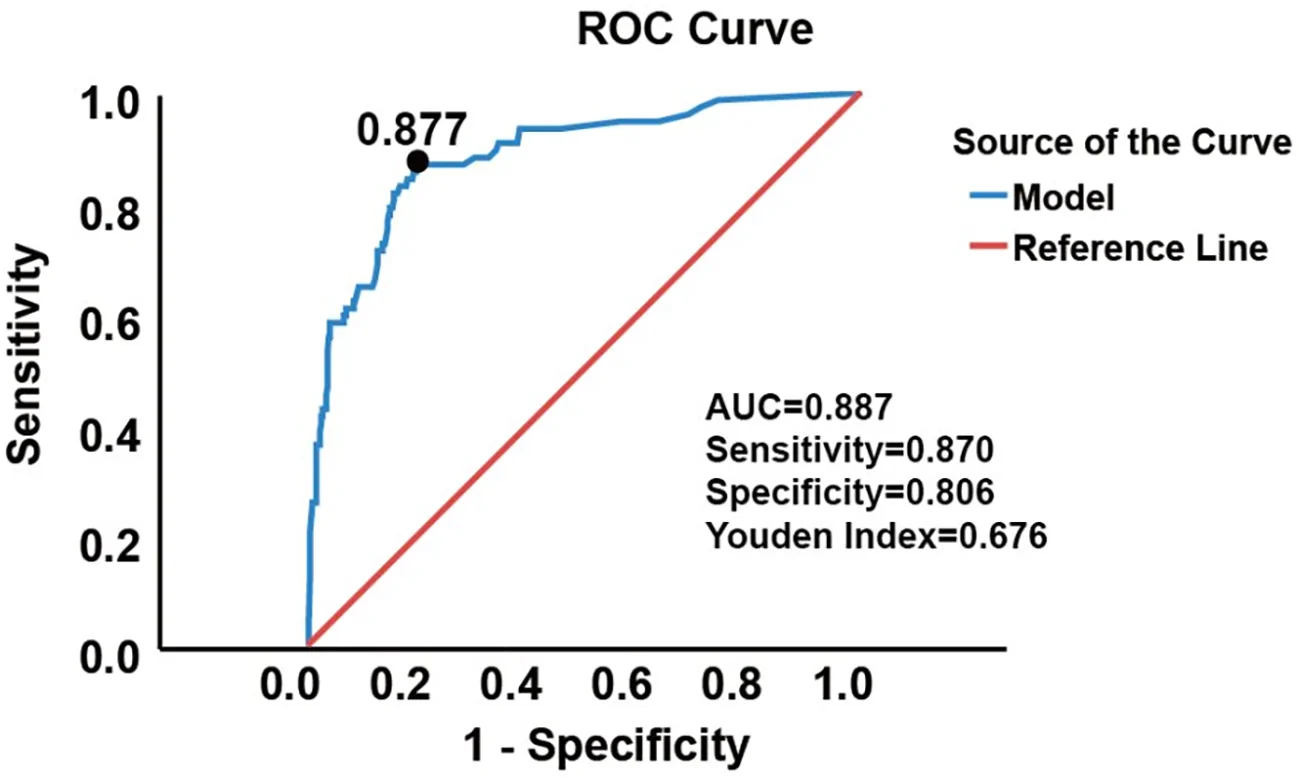
The ROC curve of the 3-year RFS rates (predicted by the nomogram model) for predicting the recurrence of EC. Description: “black dot” represents the area under the curve (AUC) at this point is the largest, which suggests that the optimal threshold of the 3-year RFS rate (risk threshold of the model) for predicting the recurrence of EC is 0.877 (area under the curve = 0.887; sensitivity, 87%; specificity, 80.6%).

In the training cohort, Kaplan–Meier analysis revealed significantly worse RFS in the high-risk group compared with the low-risk group: 3-year RFS was 58.4% (95% CI: 50.56–66.24%) versus 97.5% (95% CI: 95.93–99.07%), and 5-year RFS was 53.7% (95% CI: 45.08–62.32%) versus 97.2% (95% CI: 95.44–98.96%) (*P* < 0.001). Similarly, overall survival (OS) was markedly reduced in the high-risk group: 3-year OS was 72.3% (95% CI: 65.24–79.36%) versus 99.2% (95% CI: 98.22–100%), and 5-year OS was 64.1% (95%CI: 55.87–72.33%) versus 98.8% (95% CI: 97.62–99.98%) (*P* < 0.001). The validation cohort showed consistent findings, with significantly inferior RFS and OS in the high-risk group (**Table 3**, **Figure 7**).

**Table 3:** Analysis of survival differences between high-risk and non-high-risk group in two cohorts.

Cohort	Group	Number of recurrences	3-year RFS rate (95%CI)	5-year RFS rate (95%CI)	*P value*	Number of deaths	3-year OS rate (95%CI)	5-year OS rate (95%CI)	*P value*
Training Cohort (N = 520)	High-risk group (N = 153, 29.42%)	67(87.01%)	58.4% (50.56-66.24%)	53.7% (45.08-62.32%)	<0.001	51(92.73%)	72.3% (65.24-79.36%)	64.1% (55.87-72.33%)	<0.001
	Low-risk group (N = 367, 70.58%)	10(12.99%)	97.5% (95.93-99.07%)	97.2% (95.44-98.96%)		4(7.27%)	99.2% (98.22-100%)	98.8% (97.62-99.98%)	
Validation Cohort (N = 243)	High-risk group (N = 80, 32.92%)	32(86.49%)	63.2% (52.62-73.78%)	57.1% (45.14-69.06%)	<0.001	26(86.67%)	73.6% (64-82.2%)	65.4% (54.23-76.57%)	<0.001
	Low-risk group (N = 163, 67.08%)	5(13.51%)	96.9% (94.16-99.64%)	96.9% (94.16-99.64%)		4(13.33%)	98.8% (97.04-100%)	96.8% (93.47-100%)	

Percentages for recurrence and death were calculated using the total number of patients in each risk group as the denominator.

**Figure 7:**
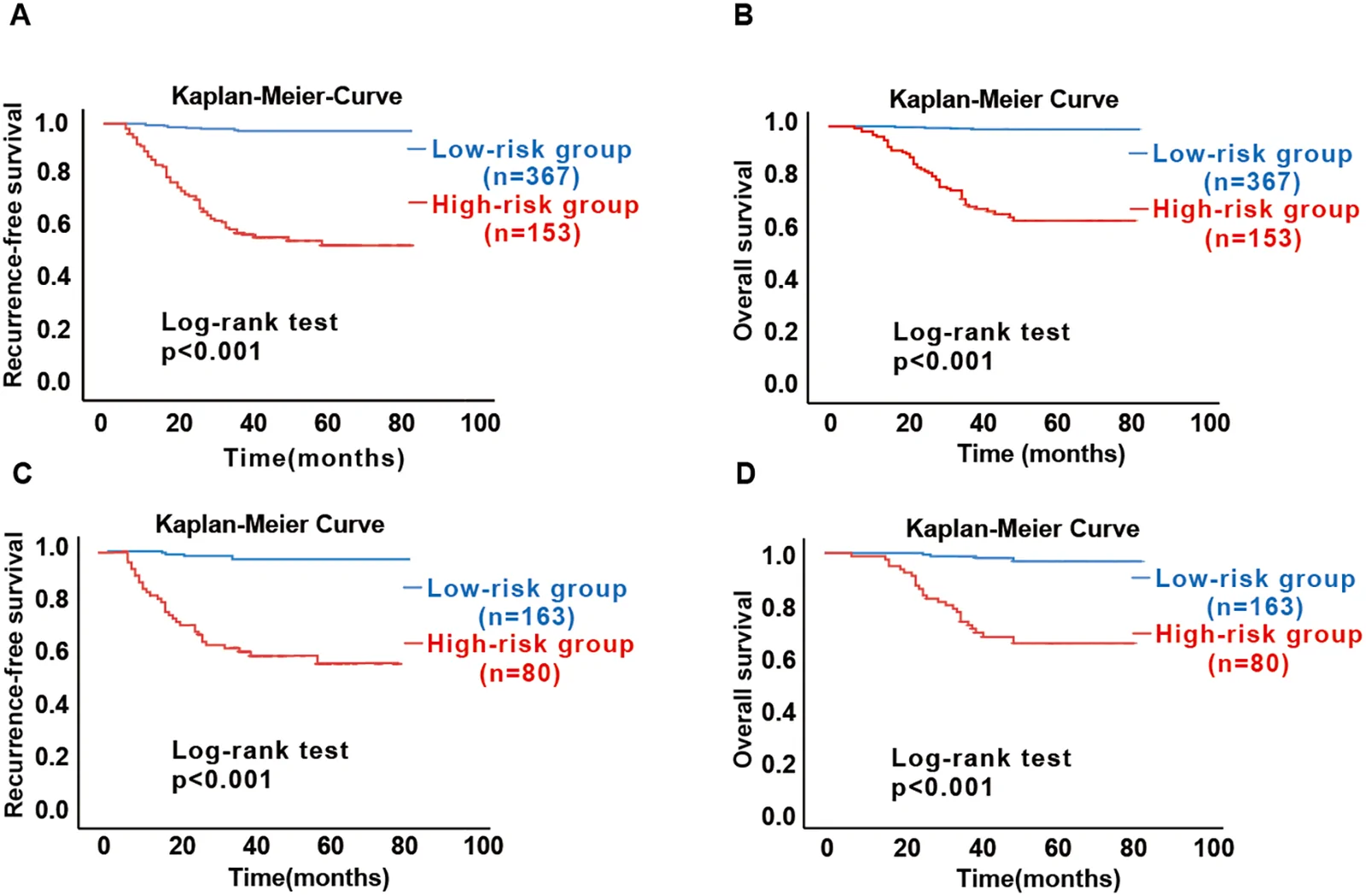
Kaplan-Meier curves depicting EC patient outcomes according to nomogram risk stratification. **(A, C)** RFS in the training and validation cohorts, stratified into high- and Low-risk groups by the model. **(B, D)** OS in the training and validation cohorts across the established high- and Low-risk categories.

### Comparison with published prediction models

The discriminative performance of our nomogram was benchmarked against three previously published EC recurrence prediction models: the FRANCOGYN model (29), the Li model (30), and the Xiao model (31), each built on clinicopathological parameters with or without molecular markers. The three models showed variable predictive performance, as quantified by the C-index. The Li model yielded the lowest C-indices (0.827 in the training set and 0.764 in the validation set), the FRANCOGYN model performed at an intermediate level (0.826 and 0.782 respectively), and the Xiao model ranked highest among the comparators (0.866 and 0.843, respectively). Our nomogram achieved C-indices of 0.892 (95%CI: 0.860–0.923) in the training cohort and 0.859 (95% CI: 0.789–0.904) in the validation cohort, both exceeding those of the three comparator models (**Table 4**).

**Table 4:** The Ppredictive performance (C-Index) of different models for predicting EC recurrence in the training cohort and validation cohort.

Model	Key predictors of the prediction model	Training cohort C-index (95% CI)	Training delong *P*†	Validation cohort C-index (95% CI)	Validation delong *P*†
Model A (29)	A nomogram including age, surgical staging, histological grade, LVSI, FIGO staging.	0.826 (0.783–0.870)	**0.027**	0.782(0.711-0.854)	0.068
Model B (30)	A nomogram including age, CA125, P53 LVSI, FIGO staging.	0.827 (0.782–0.872)	0.095	0.764 (0.690-0.838)	**0.013**
Model C (31)	A nomogram including age,histologic type, myometrial invasion, CA125,ki67 expression, ER expression, LVSI, FIGO staging, P53 status, Adjuvant treatment, HALP score	0.866 (0.832–0.901)	0.723	0.843 (0.795-0.892)	0.771
Model proposed in this study	A nomogram model including age, FIGO staging, myometrial invasion, LVSI, Histological type, P53 expression, CA125 and YTHDC2 expression	0.892 (0.858–0.926)	Reference	0.859 (0.799-0.919)	Reference

† DeLong P values are from paired time-dependent ROC (AUC) comparisons of 3-year RFS against the present nomogram. Given the high concordance between AUC and C-index for survival outcomes (C-index = 0.892 vs 3-year AUC = 0.893 in the training cohort), these P values are interpreted as approximate tests of the C-index differences.

C-index, Harrell’s concordance index. The C-index of each published model was computed by applying its published coefficients to the present cohorts. The Xiao model includes only 6 of its 11 original predictors that were available in the present dataset — age, histological type, myometrial invasion, FIGO stage, LVSI, and p53 status. The remaining 5 predictors (Ki-67, ER, molecular classification, adjuvant therapy, HALP score) were unavailable and treated as reference level, which may underestimate Xiao’s discriminative ability.

Bold values indicate P < 0.05.

## Discussion

This multicenter study developed and externally validated a nomogram integrating YTHDC2 expression with clinicopathological parameters for predicting RFS in patients with EC. High YTHDC2 expression, assessed by IHC, proved to be an independent protective factor against EC recurrence (HR = 0.26, *P* < 0.001). The combined nomogram achieved an AUC of 0.887 in the training cohort, surpassing both YTHDC2 alone (AUC = 0.680) and a clinicopathology-only model (AUC = 0.823). Risk stratification derived from the nomogram effectively discriminated high-risk from low-risk patients, with significant separation in both 3-year RFS and OS (**Figure 7**).

We further compared our model with three published EC prediction models (**Table 4**). In the training and validation sets of this study, the C-index was 0.826 and 0.782 for the FRANCOGYN model (29), 0.827 and 0.764 for the Li model (30), and 0.866 and 0.843 for the Xiao model (31). Our model attained C-indices of 0.892 and 0.859, exceeding all three comparator models in both cohorts. In the validation cohort, our model retained the highest C-index (0.859) among all four models despite a moderate decrease from training (Δ = 0.034); by contrast, the Li model showed the largest drop (Δ = 0.063) and the FRANCOGYN model a substantial one (Δ = 0.045). Although the Xiao model showed the smallest decrease (Δ = 0.023), its C-index in both cohorts remained below that of our nomogram. We attribute this advantage to the epitranscriptomic dimension contributed by YTHDC2, which captures prognostic information that traditional clinicopathological parameters and inflammatory indicators do not, thereby helping the model maintain discriminative capacity across different populations.

YTHDC2, an m^6^A reader protein, governs RNA splicing, stability, and translation (32, 33). Its tumor-suppressive role has been reported in bladder (34), hepatocellular (35), and colorectal cancers (36), primarily via m^6^A-dependent post-transcriptional regulation. Our findings are consistent with this evidence: high YTHDC2 expression was independently associated with reduced EC recurrence (HR = 0.26, *P* < 0.001). Although the precise molecular mechanism by which YTHDC2 suppresses EC recurrence awaits experimental elucidation, the clinical data presented here provide robust evidence that YTHDC2 expression carries independent prognostic value and can inform risk stratification.

Incorporating YTHDC2 into the clinicopathological model yielded a meaningful improvement in discriminative accuracy (ΔAUC=+0.064). Although this absolute increment appears modest, every marginal gain in prognostic modeling carries clinical weight. This improvement suggests that YTHDC2 captures prognostic information not fully reflected by conventional parameters. Notably, YTHDC2 alone showed limited discrimination (AUC = 0.680), underscoring that EC recurrence is a multifactorial process that no single biomarker can adequately predict. The superior performance of the combined model reinforces the principle — and practical advantage — of integrating molecular markers with established clinicopathological variables.

FIGO stage, depth of myometrial invasion, and LVSI were confirmed as independent predictors of RFS, underscoring the importance of tumor anatomical extent in recurrence risk assessment (37, 38). Age ≥60 years and elevated CA125 (≥35 U/mL) were also independently linked to worse prognosis, in agreement with large-cohort data (39). p53 IHC status was among the factors retained in the model. Although the 2023 FIGO system has incorporated molecular subtypes — including p53 mutational status — into EC risk stratification (13), comprehensive genomic sequencing is not routinely accessible across many clinical settings. In line with Wang, Zheng, and Jiang et al (40), our data suggest that p53 IHC can function as a practical surrogate for molecular classification, yielding prognostic information in the absence of next-generation sequencing.

YTHDC2 and p53 showed no significant association and displayed distinct subcellular localization (cytoplasmic vs. nuclear), suggesting they capture complementary prognostic information through independent pathways. Whereas p53 IHC largely reflects TP53 mutational status, YTHDC2 — an m^6^A reader — reports on epitranscriptomic regulation. Their independence strengthens the rationale for integrating both markers into the nomogram.

The nomogram-based risk stratification separated patients into two groups with sharply different RFS and OS at 1, 3, and 5 years. Clinically, this has concrete implications: high-risk patients may warrant closer surveillance, reconsideration of adjuvant therapy, and tighter follow-up, whereas low-risk patients could avoid overtreatment and its harms. All components of the nomogram — YTHDC2 IHC, p53 IHC, and standard clinicopathological parameters — are obtainable through routine pathology practice. YTHDC2 IHC, in particular, is straightforward, inexpensive, and within reach for primary-care hospitals where molecular testing capacity is limited.

The study has several strengths. It is, to the best of our knowledge, the first to evaluate YTHDC2 as a prognostic biomarker and incorporate it into an EC nomogram. The multicenter design with independent external validation (training cohort, n = 520; validation cohort, n = 243) strengthens the reliability of the results, and the use of IHC for both YTHDC2 and p53 makes the model broadly applicable in clinical practice.

Several limitations warrant comment. The retrospective design introduces inevitable selection bias. The three validation centres are located within the same geographic region (Chongqing/Sichuan), and immunohistochemistry was performed centrally, which limits the population and technical diversity of a fully independent external validation. Centralised staining ensures consistent scoring, but complementary validation in geographically distinct cohorts would further strengthen generalisability. We did not perform transcriptomic validation (e.g., TCGA), because our nomogram relies on protein-level immunohistochemical inputs that cannot be recapitulated from mRNA data, and because the transcriptomic prognostic value of YTHDC2 has already been reported by prior TCGA-based studies. Comprehensive genomic profiling — including POLE mutation and mismatch-repair status — was unavailable for most patients, precluding a direct examination of how YTHDC2 interacts with the established molecular subtypes; although p53 IHC served as a practical surrogate in our nomogram, it does not fully capture these subtypes, whose prognostic relevance is well established (13). Our correlation analysis nonetheless showed YTHDC2 and p53 to be essentially independent (Spearman ρ = 0.020, *P* = 0.645), suggesting complementary rather than redundant prognostic information. YTHDC2 was quantified by semiquantitative IHC alone, and mass spectrometry–based proteomics may offer finer resolution in future studies. In a competing-risk analysis treating non-cancer death as a competing event, subdistribution hazard ratios were consistent with the main Cox results (**Supplementary Table 2**), indicating that our conclusions are robust in this setting.

Prospective validation in geographically diverse cohorts incorporating contemporary molecular classification will be important before the nomogram can be considered for clinical decision-making.

Several lines of work follow from these findings. Multi-omics approaches— combining m^6^A sequencing (MeRIP-seq), RNA-seq, and proteomics—could pinpoint the YTHDC2-regulated transcripts and signaling pathways that mediate its protective function. Adding other m^6^A regulators (METTL3, METTL14, FTO, ALKBH5) to the nomogram may further improve its discriminative ability. Whether YTHDC2 expression shapes response to immunotherapy or targeted agents also warrants investigation, as it may point toward individualized treatment strategies in EC.

## Conclusions

This multicenter study establishes YTHDC2 as an independent protective factor against EC recurrence. Combining YTHDC2 with clinicopathological parameters in a nomogram yielded more accurate RFS prediction and risk stratification than single-component alternatives. This nomogram may offer a practical, accessible tool for individualized postoperative management and surveillance in EC.

## Data Availability

The original contributions presented in the study are included in the article/**Supplementary Material**. Further inquiries can be directed to the corresponding authors.
