# Supplementary material for: A nomogram integrating YTHDC2 expression and clinicopathological parameters for predicting endometrial cancer recurrence: a multicenter retrospective study
Source: Front Oncol. 2026 Oct 19;16:1930179. doi: 10.3389/fonc.2026.1930179 (PMC13724511; doi:10.3389/fonc.2026.1930179)
Supplement: Supplementary file 1 [file DataSheet1.pdf]

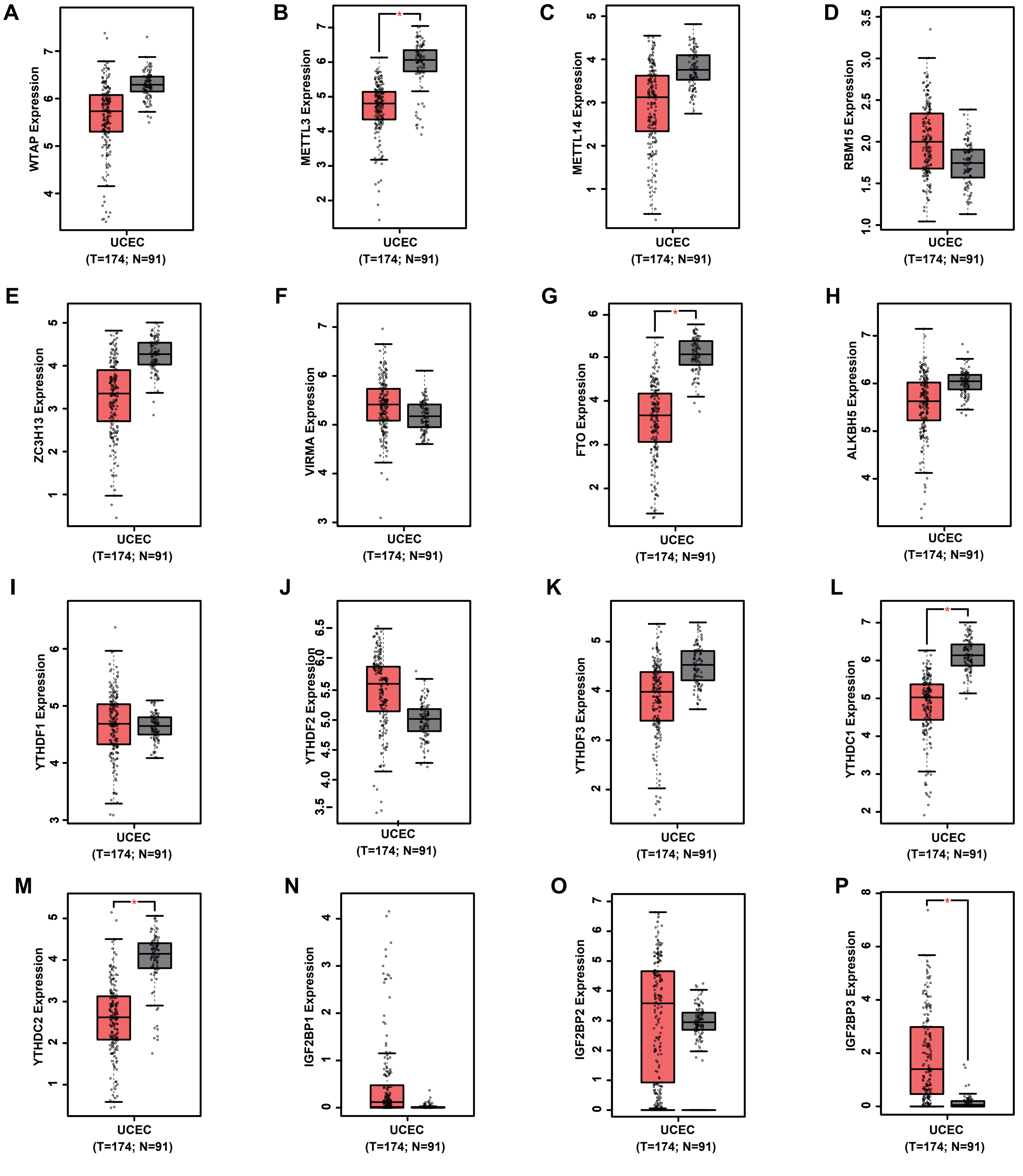

Supplementary figure 1: GEPIA database was employed to analyze the expression patterns of 16 m6A-related genes across EC and normal tissue specimens.
