# Supplementary material for: A nomogram integrating YTHDC2 expression and clinicopathological parameters for predicting endometrial cancer recurrence: a multicenter retrospective study
Source: Front Oncol. 2026 Oct 19;16:1930179. doi: 10.3389/fonc.2026.1930179 (PMC13724511; doi:10.3389/fonc.2026.1930179)
Supplement: Supplementary file 2 [file DataSheet2.pdf]

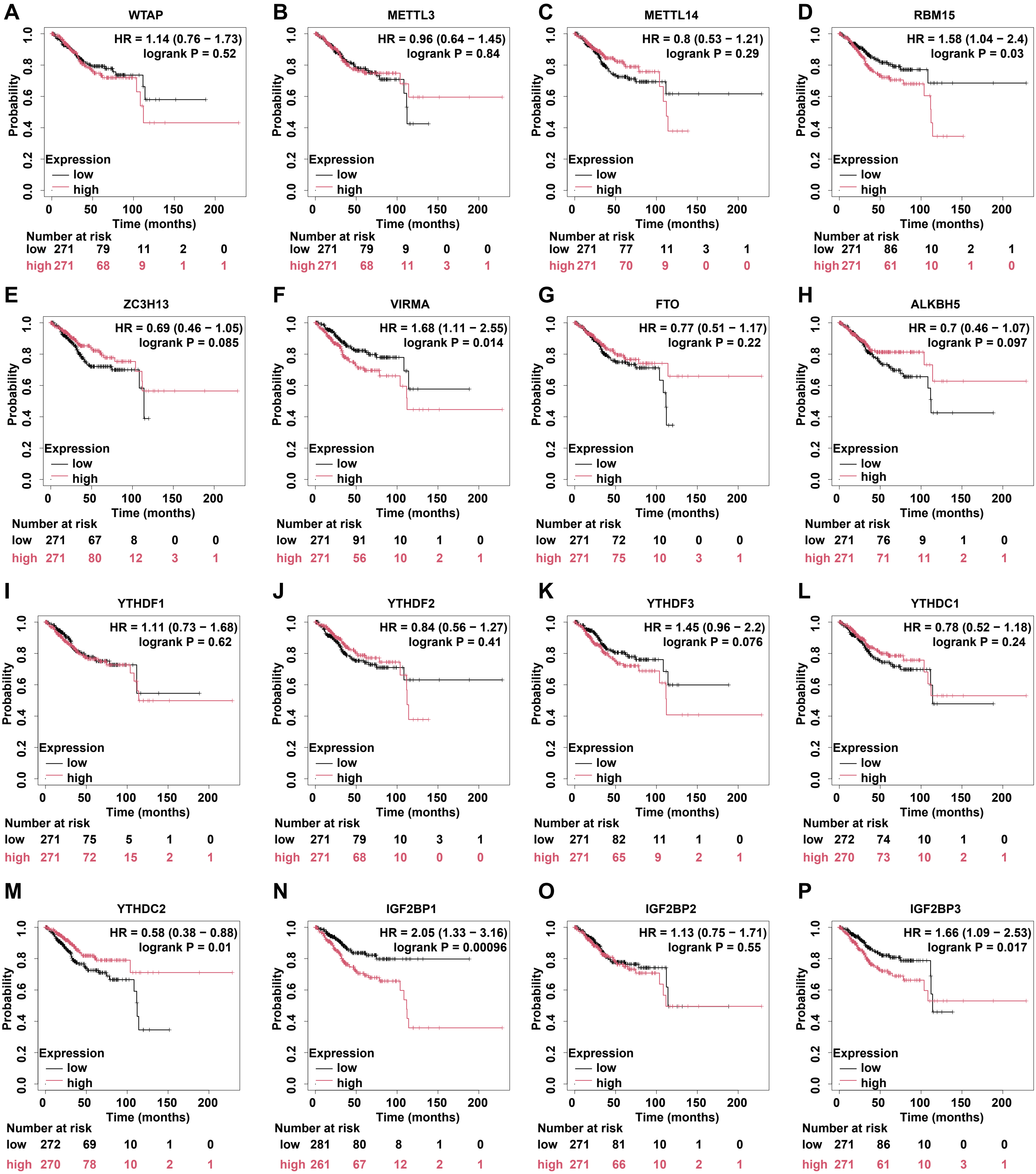


**Supplementary figure 2: Overall survival (OS) curves for 16 m6A-related genes in EC were generated through the KM Plotter database, comparing EC patients with high (red) versus low (black) expression levels.**
