# Supplementary material for: A nomogram integrating YTHDC2 expression and clinicopathological parameters for predicting endometrial cancer recurrence: a multicenter retrospective study
Source: Front Oncol. 2026 Oct 19;16:1930179. doi: 10.3389/fonc.2026.1930179 (PMC13724511; doi:10.3389/fonc.2026.1930179)
Supplement: Supplementary file 3 [file DataSheet3.pdf]

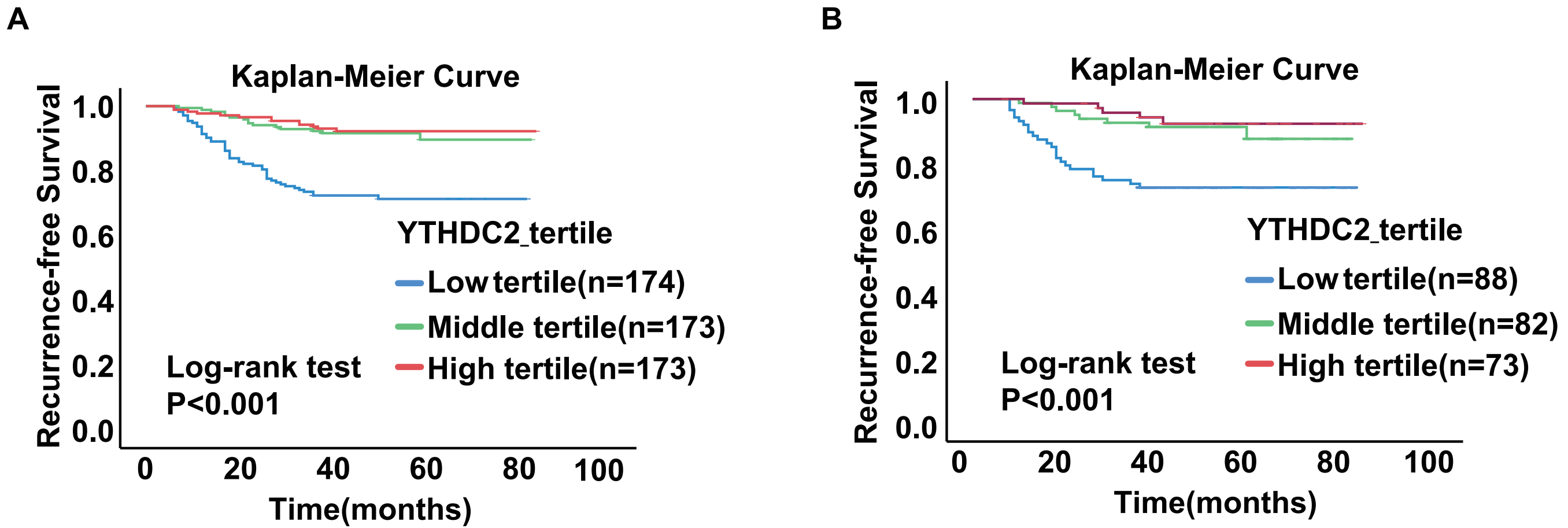

Supplementary Figure 3. Kaplan–Meier curves for recurrence-free survival (RFS) stratified by YTHDC2 expression tertiles in the training (A) and validation (B) cohorts. The continuous YTHDC2 immunohistochemistry score was categorized into tertiles (Low, Middle, High) using cutpoints derived from the training cohort (P33 = 2.47, P67 = 3.16), which were then applied to the validation cohort. The number of patients in each tertile is shown in the legend (n = 174/173/173 for training; n = 88/82/73 for validation). A significant dose–response relationship was observed, with the Low tertile showing the poorest 3-year RFS (training: 72.4%; validation: 72.7%), and the Middle and High tertiles showing substantially better 3-year RFS (training: 92.4% and 93.6%; validation: 92.7% and 94.3%). P values were calculated using the two-sided log-rank test (training: $P < 0.001$; validation: $P < 0.001$). RFS, recurrence-free survival.
