# Supplementary material for: A nomogram integrating YTHDC2 expression and clinicopathological parameters for predicting endometrial cancer recurrence: a multicenter retrospective study
Source: Front Oncol. 2026 Oct 19;16:1930179. doi: 10.3389/fonc.2026.1930179 (PMC13724511; doi:10.3389/fonc.2026.1930179)
Supplement: Supplementary file 4 [file DataSheet4.pdf]

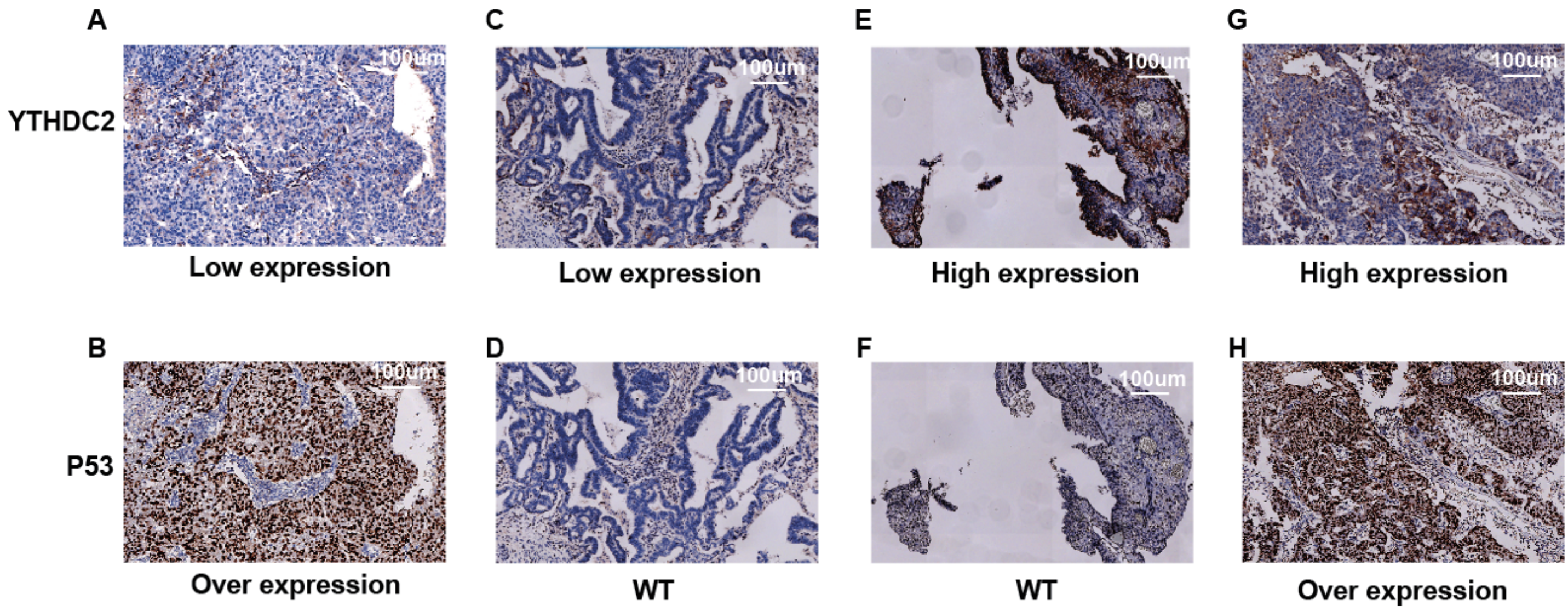

Supplymentary Figure 4: **paired serial sections showing YTHDC2 (top, cytoplasmic) and p53 (bottom, nuclear) immunostaining from the same tumour areas. Four representative endometrial carcinomas are shown (A–B, C–D, E–F, G–H), illustrating all four combinations of YTHDC2 expression (low/high) and p53 status (wild-type/overexpression). The co-occurrence of YTHDC2-low with both p53-wild-type (C–D) and p53-overexpression (A–B), and YTHDC2-high with both p53-wild-type (E–F) and p53-overexpression (G–H), demonstrates the independence of YTHDC2 and p53 within the same tumour.**
