# Supplementary material for: A nomogram integrating YTHDC2 expression and clinicopathological parameters for predicting endometrial cancer recurrence: a multicenter retrospective study
Source: Front Oncol. 2026 Oct 19;16:1930179. doi: 10.3389/fonc.2026.1930179 (PMC13724511; doi:10.3389/fonc.2026.1930179)
Supplement: Supplementary file 5 [file DataSheet5.pdf]

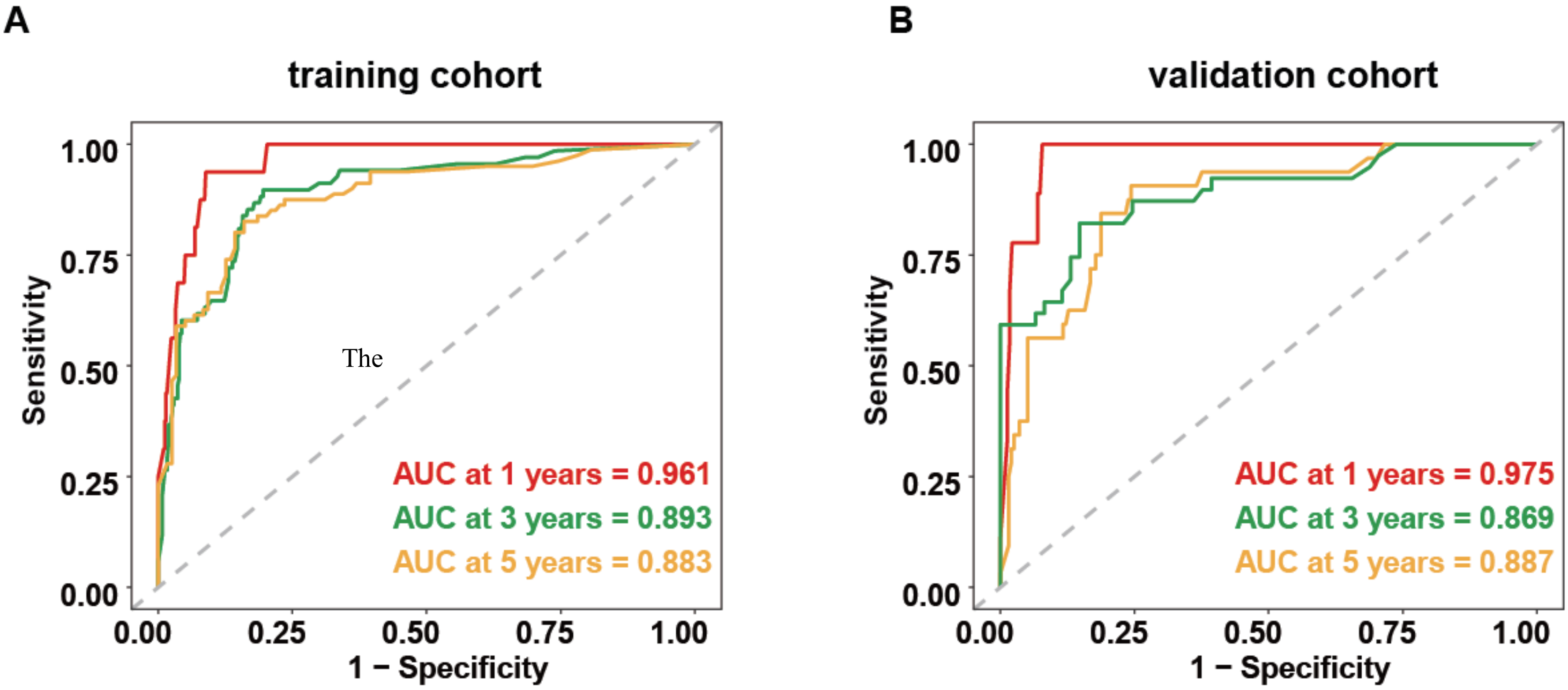

Supplymentary Figure 5: **The ROC curves of the nomogram. The ROC curves of the model were used to predict 1-, 3-, 5-year RFS probability in the training cohort (A) and validation cohort (B)**
