# Supplementary material for: A nomogram integrating YTHDC2 expression and clinicopathological parameters for predicting endometrial cancer recurrence: a multicenter retrospective study
Source: Front Oncol. 2026 Oct 19;16:1930179. doi: 10.3389/fonc.2026.1930179 (PMC13724511; doi:10.3389/fonc.2026.1930179)
Supplement: Supplementary file 6 [file DataSheet6.pdf]

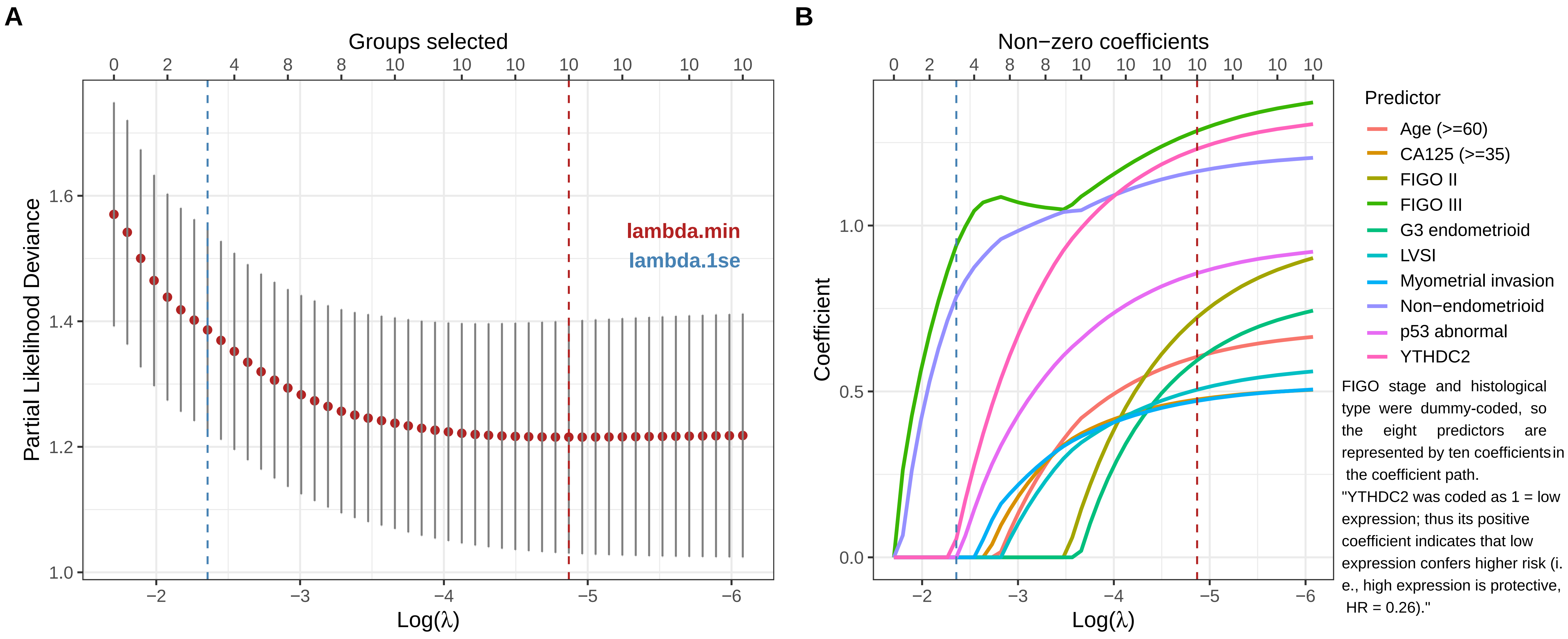
A
Groups selected
0 2 4 8 8 10 10 10 10 10 10 10
Partial Likelihood Deviance
1.0
1.2
1.4
1.6
lambda.min
lambda.1se
−2 −3 −4 −5 −6
Log(λ)
B
Non−zero coefficients
0 2 4 8 8 10 10 10 10 10 10 10
Coefficient
0.0
0.5
1.0
−2 −3 −4 −5 −6
Log(λ)
Predictor
Age (>=60)
CA125 (>=35)
FIGO II
FIGO III
G3 endometrioid
LVSI
Myometrial invasion
Non−endometrioid
p53 abnormal
YTHDC2
FIGO stage and histological type were dummy-coded, so the eight predictors are represented by ten coefficients in the coefficient path.
"YTHDC2 was coded as 1 = low expression; thus its positive coefficient indicates that low expression confers higher risk (i.e., high expression is protective, HR = 0.26)."
