# Supplementary material for: A nomogram integrating YTHDC2 expression and clinicopathological parameters for predicting endometrial cancer recurrence: a multicenter retrospective study
Source: Front Oncol. 2026 Oct 19;16:1930179. doi: 10.3389/fonc.2026.1930179 (PMC13724511; doi:10.3389/fonc.2026.1930179)
Supplement: Supplementary file 7 [file DataSheet7.pdf]

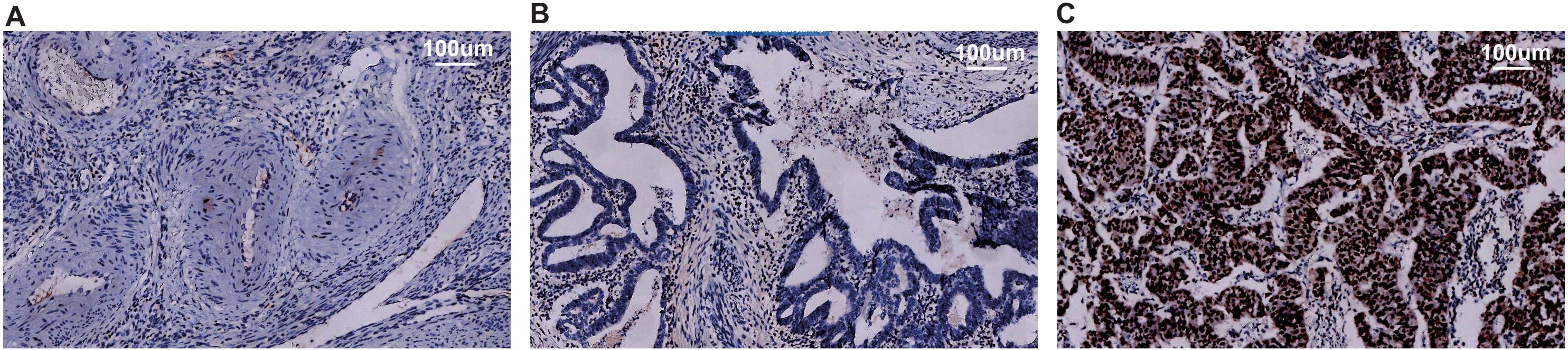

Supplymentary Figure 7:(A) Normal wild-type pattern: an admixture of negative, weakly, and strongly positive tumour nuclei with variable staining intensity. (B) Complete absence (null pattern): no nuclear staining in tumour cells, with internal positive controls (stromal fibroblasts and lymphocytes) showing intact nuclear staining, confirming technical adequacy. (C) Overexpression (mutation-type pattern): diffuse strong nuclear staining in ≥80% of tumour cells. Both patterns (B) and (C) were classified as aberrant/mutation-type.
