# Supplementary material for: A nomogram integrating YTHDC2 expression and clinicopathological parameters for predicting endometrial cancer recurrence: a multicenter retrospective study
Source: Front Oncol. 2026 Oct 19;16:1930179. doi: 10.3389/fonc.2026.1930179 (PMC13724511; doi:10.3389/fonc.2026.1930179)
Supplement: Supplementary file 8 [file Table1.docx]

**Supplementary Table 1. Schoenfeld residual test of the proportional hazards assumption for the final multivariable Cox model (training cohort)**

| **Covariate** | **χ²** | **df** | ***P*** |
| --- | --- | --- | --- |
| Age ≥60 years | 0.19 | 1 | 0.66 |
| FIGO stage | 0.53 | 2 | 0.77 |
| Myometrial invasion ≥1/2 | 0.09 | 1 | 0.77 |
| LVSI positive | 0.14 | 1 | 0.71 |
| Histological type | 0.61 | 2 | 0.74 |
| CA125 >35 U/mL | 0.65 | 1 | 0.42 |
| p53 abnormal | 0.09 | 1 | 0.76 |
| YTHDC2 high expression | 0.01 | 1 | 0.91 |
| **Global** | **2.49** | **10** | **0.99** |

χ², chi-square statistic; df, degrees of freedom.

The proportional hazards assumption was assessed with the cox.zph function (survival package, R). All individual *P* > 0.05 and global *P* = 0.99 indicate no violation of the proportional hazards assumption.
