# Supplementary material for: A nomogram integrating YTHDC2 expression and clinicopathological parameters for predicting endometrial cancer recurrence: a multicenter retrospective study
Source: Front Oncol. 2026 Oct 19;16:1930179. doi: 10.3389/fonc.2026.1930179 (PMC13724511; doi:10.3389/fonc.2026.1930179)
Supplement: Supplementary file 9 [file Table2.docx]

**Supplementary Table 2. Comparison of main Cox and Fine-Gray competing-risk hazard ratios for recurrence-free survival in the training cohort (n = 520)**

| **Predictor** | **Main Cox HR (95% CI)** | **Cox *P*** | **Fine-Gray sHR (95% CI)** | **Fine-Gray *P*** |
| --- | --- | --- | --- | --- |
| Age (≥60 vs. <60) | 2.03 (1.24–3.32) | 0.005 | 1.99 (1.23–3.22) | 0.005 |
| FIGO stage II (vs. I) | 2.67 (1.14–6.25) | 0.024 | 2.68 (1.11–6.44) | 0.028 |
| FIGO stage III (vs. I) | 4.16 (2.07–8.36) | <0.001 | 4.10 (2.01–8.35) | <0.001 |
| Myometrial invasion (≥1/2 vs. <1/2) | 1.69 (1.03–2.75) | 0.037 | 1.69 (1.04–2.72) | 0.033 |
| LVSI (present vs. absent) | 1.84 (1.11–3.05) | 0.018 | 1.81 (1.13–2.90) | 0.013 |
| G3 endometrioid (vs. G1/G2) | 2.28 (1.03–5.05) | 0.042 | 2.27 (1.02–5.05) | 0.044 |
| Non-endometrioid (vs. G1/G2) | 3.47 (1.98–6.07) | <0.001 | 3.44 (1.92–6.14) | <0.001 |
| CA125 (>35 vs. ≤35 U/mL) | 1.68 (1.03–2.74) | 0.037 | 1.67 (1.07–2.60) | 0.024 |
| p53 (aberrant vs. normal) | 2.59 (1.57–4.27) | <0.001 | 2.61 (1.61–4.25) | <0.001 |
| YTHDC2 (high vs. low expression) | 0.25 (0.15–0.42) | <0.001 | 0.26 (0.16–0.43) | <0.001 |

Footnotes:HR, hazard ratio; sHR, subdistribution hazard ratio; CI, confidence interval.

Reference categories: age <60 years, FIGO stage I, myometrial invasion <1/2, LVSI absent, G1/G2 endometrioid, CA125 ≤35 U/mL, p53 normal, YTHDC2 low expression.

YTHDC2 high expression was independently protective: Cox HR = 0.25 (0.15–0.42), Fine-Gray sHR = 0.26 (0.16–0.43), both *P* < 0.001.

The Fine-Gray model treated death without prior recurrence as the competing event; only 1 patient (0.19%) died without recurrence, so subdistribution hazard ratios were nearly identical to the main Cox estimates.
