# Supplementary material for: A nomogram integrating YTHDC2 expression and clinicopathological parameters for predicting endometrial cancer recurrence: a multicenter retrospective study
Source: Front Oncol. 2026 Oct 19;16:1930179. doi: 10.3389/fonc.2026.1930179 (PMC13724511; doi:10.3389/fonc.2026.1930179)
Supplement: Supplementary file 10 [file Table3.docx]

| **Cohort** | **YTHDC2 tertile** | **n** | **Recurrences, n (%)** | **3-year RFS, %** |
| --- | --- | --- | --- | --- |
| Training (n = 520) | Low (< 2.47) | 174 | 49 (28.2) | 72.4 |
|  | Middle (2.47–3.16) | 173 | 15 (8.7) | 92.4 |
|  | High (≥ 3.16) | 173 | 13 (7.5) | 93.6 |
|  | *P* value (log-rank) | - | - | < 0.001 |
|  | *P* value (trend) | - | - | < 0.001 |
| Validation (n = 243) | Low (< 2.47) | 88 | 24 (27.3) | 72.7 |
|  | Middle (2.47–3.16) | 82 | 8 (9.8) | 92.7 |
|  | High (≥ 3.16) | 73 | 5 (6.8) | 94.3 |
|  | *P* value (log-rank) | - | - | < 0.001 |
|  | *P* value (trend) | - | - | < 0.001 |

**Supplementary Table 3. Sensitivity analysis of the YTHDC2 cutoff using tertiles of the continuous YTHDC2 score.**

Footnote:The tertile cutpoints (2.47 and 3.16) were derived from the training cohort and applied to both cohorts. Log-rank test (df = 2): training,χ²= 38.35; validation,χ²= 16.80.Test for linear trend (df = 1): training,χ²= 26.72; validation, χ²= 12.53. RFS, recurrence-free survival.
