# Supplementary material for: A nomogram integrating YTHDC2 expression and clinicopathological parameters for predicting endometrial cancer recurrence: a multicenter retrospective study
Source: Front Oncol. 2026 Oct 19;16:1930179. doi: 10.3389/fonc.2026.1930179 (PMC13724511; doi:10.3389/fonc.2026.1930179)
Supplement: Supplementary file 11 [file Table4.docx]

| YTHDC2 expression | p53 normal, n (%) | p53 aberrant, n (%) | Total, n (%) |
| --- | --- | --- | --- |
| High | 243 (71.1) | 99 (28.9) | 342 (100.0) |
| Low | 123 (69.1) | 55 (30.9) | 178 (100.0) |
| Total | 366 (70.4) | 154 (29.6) | 520 (100.0) |

**Supplementary Table 4：Cross-tabulation of YTHDC2 and p53 expression in the training cohort**

Note:Percentages are row percentages. Chi-square test: *P* = 0.645. Spearman's rank correlation: ρ = 0.020, *P* = 0.645. All expected cell counts exceeded 5 (minimum 52.7). Abbreviations: p53, tumour protein p53.
