# Supplementary material for: A nomogram integrating YTHDC2 expression and clinicopathological parameters for predicting endometrial cancer recurrence: a multicenter retrospective study
Source: Front Oncol. 2026 Oct 19;16:1930179. doi: 10.3389/fonc.2026.1930179 (PMC13724511; doi:10.3389/fonc.2026.1930179)
Supplement: Supplementary file 12 [file Table5.docx]

**Supplementary Table 5. Variance inflation factors for the predictors in the final Cox model**

| **Predictors** | **VIF** |
| --- | --- |
| Age (≥60 vs. <60 years) | 1.20 |
| FIGO stage (I / II / III) | 2.05 |
| Myometrial invasion (<1/2 vs. ≥1/2) | 1.17 |
| LVSI (Negative VS Positive) | 1.25 |
| Histological type (G1/G2, G3, non-endometrioid) | 1.49 |
| CA125 (≤35 vs. >35 U/mL) | 1.18 |
| p53 (normal vs. abnormal) | 1.15 |
| YTHDC2 (high vs. low) | 1.07 |

Note: VIF, variance inflation factor. For multi-category variables (FIGO stage and histological type), the maximum VIF across categories is reported. All VIF values are below 5, indicating no significant multicollinearity.
